# Accurate and Reliable Cancer Classification Based on Probabilistic Inference of Pathway Activity

**DOI:** 10.1371/journal.pone.0008161

**Published:** 2009-12-07

**Authors:** Junjie Su, Byung-Jun Yoon, Edward R. Dougherty

**Affiliations:** 1 Department of Electrical and Computer Engineering, Texas A&M University, College Station, Texas, United States of America; 2 Computational Biology Division, Translational Genomics Research Institute, Phoenix, Arizona, United States of America; IBM Thomas J. Watson Research Center, United States of America

## Abstract

With the advent of high-throughput technologies for measuring genome-wide expression profiles, a large number of methods have been proposed for discovering diagnostic markers that can accurately discriminate between different classes of a disease. However, factors such as the small sample size of typical clinical data, the inherent noise in high-throughput measurements, and the heterogeneity across different samples, often make it difficult to find reliable gene markers. To overcome this problem, several studies have proposed the use of pathway-based markers, instead of individual gene markers, for building the classifier. Given a set of known pathways, these methods estimate the activity level of each pathway by summarizing the expression values of its member genes, and use the pathway activities for classification. It has been shown that pathway-based classifiers typically yield more reliable results compared to traditional gene-based classifiers. In this paper, we propose a new classification method based on probabilistic inference of pathway activities. For a given sample, we compute the log-likelihood ratio between different disease phenotypes based on the expression level of each gene. The activity of a given pathway is then inferred by combining the log-likelihood ratios of the constituent genes. We apply the proposed method to the classification of breast cancer metastasis, and show that it achieves higher accuracy and identifies more reproducible pathway markers compared to several existing pathway activity inference methods.

## Introduction

The introduction of affordable microarray technologies for measuring genome-wide expression profiles has led to the development of numerous methods for discriminating between different classes of a complex disease, such as cancer, through transcriptome analysis [1]–[4]. Especially, there have been significant research efforts to identify differentially expressed genes across different phenotypes [5]–[9], which can be used as diagnostic markers for classifying the disease states or predicting the outcome of medical treatments [1]–[4], [10]–[12]. However, finding reliable gene markers is a challenging problem, and several recent studies have questioned the reliability of many classifiers based on individual gene markers [13]–[19]. The small sample size of typical clinical data that are used to build a classifier is one of the major factors that make this problem difficult. We often have to search for a small number of good marker genes among thousands of genes based on a limited number of samples, which makes the performance of traditional feature selection methods quite unpredictable [20]. The inherent measurement noise in high-throughput experimental data and the heterogeneity across samples and patients make the problem even more formidable.

One possible way to address this problem is to interpret the expression data at the level of functional modules, such as signaling pathways and molecular complexes, instead of at the level of individual genes. In fact, one of the weaknesses of many gene-based classification methods is that the marker genes are often selected independently, even though their functional products may interact with each other. Therefore, the selected gene markers may contain redundant information, and they may not synergistically improve the overall classification performance. We can alleviate this problem by jointly analyzing the expression levels of groups of functionally related genes, which can be obtained based on transcriptome analysis [21]–[23], GO annotations [24], or other sources. In fact, several studies [23], [25]–[28] have shown that pathway markers are more reproducible compared to single gene markers and they can provide important biological insights into the underlying mechanisms that lead to different disease phenotypes. Furthermore, pathway-based classifiers often achieve comparable or better classification performance compared to traditional gene-based classifiers.

To use pathway-based markers in classification, we need a way to infer the activity of a given pathway based on the expression levels of the constituent genes. Recently, a number of pathway activity inference methods have been proposed for this purpose. For example, Guo et al. [25] proposed to use the mean or median expression value of the member genes to infer the pathway activity. Tomfohr et al. [28] and Bild et al. [23] used the first principal component of the expression profile of the member genes to estimate the activity of a given pathway. More recently, Lee et al. [26] proposed a method that predicts the pathway activity using only a subset of genes in the pathway, called the condition-responsive genes (CORGs), whose combined expression levels can accurately discriminate the phenotypes of interest.

In this paper, we propose a novel method for probabilistic inference of pathway activities. For a given pathway, the proposed method estimates the log-likelihood ratio between different phenotypes based on the expression level of each member gene. The activity level of the pathway is then inferred by combining the log-likelihood ratios of the genes that belong to the pathway. We apply our method to the classification of breast cancer metastasis, and demonstrate that it can achieve higher accuracy compared to several previous pathway-based approaches. Furthermore, we show that the proposed pathway activity inference method can find more reproducible pathway markers that retain the discriminative power across different datasets.

## Methods

### Datasets

We obtained two independent breast cancer datasets from large-scale gene expression studies by Wang et al. [11] (referred as the “USA” dataset in this work) and van't Veer et al. [10] (referred as the “Netherlands” dataset). Wang et al.'s dataset [11] contains the gene expression profiles of 286 breast cancer patients from the USA, where metastasis was detected in 107 of them while the remaining 179 were metastasis-free. The other dataset studied by van't Veer et al. [10] contains the gene expression profiles of 295 patients from the Netherlands, where 79 had metastasis and 216 were metastasis-free. In this study, we did not consider the follow-up time or the occurrence of distant metastasis.

To obtain the set of known biological pathways, we referred to the MSigDB (Molecular Signatures Database) version 2.4 (updated April 7, 2008) [21]. We downloaded the canonical pathways in the C2 curated gene sets, which contains 639 gene sets obtained from several pathway databases, including the KEGG (Kyoto Encyclopedia of Genes and Genomes) database [29] and the GenMAPP [30]. These gene sets are compiled by domain experts and they provide canonical representations of biological processes. The set of pathways obtained from the MSigDB covers more than 5,000 distinct genes, where 3,271 of them can be found in both microarray platforms used by the two breast cancer gene expression studies in [10], [11].

### Probabilistic Inference of Pathway Activity

For each pathway, we first identified the genes that were included in the expression profiles in the two breast cancer datasets. The genes that were not included in these datasets were removed from the gene set for the given pathway. Consider a pathway that contains 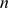 genes 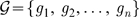 after removing the genes whose expression values were not available. Given a sample 
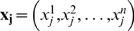
 that contains the expression levels of the member genes, we estimate the pathway activity 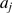 as follows
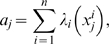
(1)where 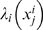 is the log-likelihood ratio (LLR) between the two phenotypes of interest for the gene 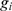. The LLR 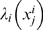 is given by
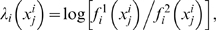
(2)where 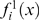 is the conditional probability density function (PDF) of the expression level of gene 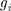 under phenotype 1, and 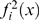 is the conditional PDF under phenotype 2. The ratio 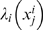 is a probabilistic indicator that tells us which phenotype is more likely based on the expression level 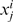 of the 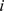
th member gene 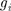. We combine the evidence from all the member genes to infer the overall pathway activity 
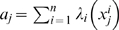
. The pathway activity 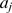 can serve as a discriminative score for classifying the sample 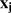 into different phenotypes based on the activation level of the given pathway. Conceptually, we can view this approach as computing the relative support for the two different phenotypes using a Naive Bayes model [31], [32] based on the gene expression profile of the pathway.

**Figure 1:**
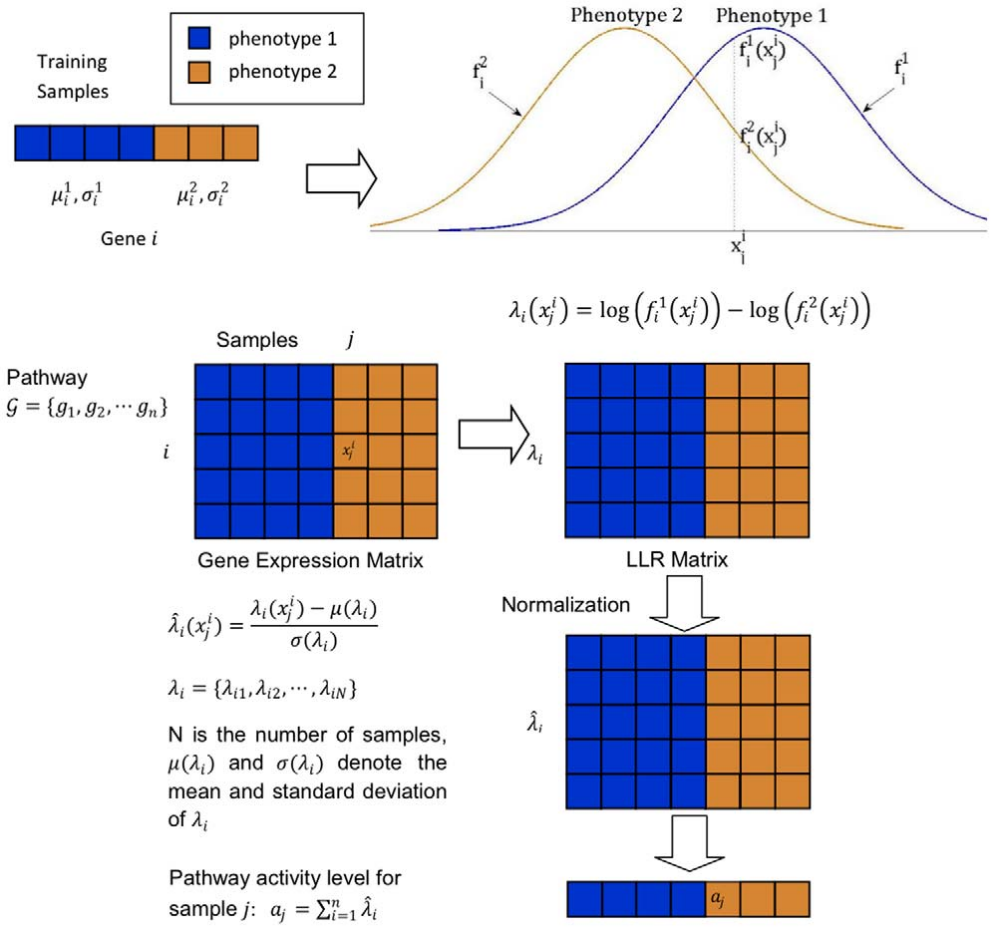
Probabilistic inference of pathway activity. For each gene in the pathway, we estimate the conditional probability density functions (PDFs) under different phenotypes. Based on the estimated PDFs, we transform the expression values of the member genes into log-likelihood ratios (LLRs) to obtain a LLR matrix from the gene expression matrix. The LLR matrix is then normalized, and the pathway activity is inferred by combining the normalized LLRs of its member genes.

In order to compute the LLR value 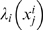, we need to estimate the PDF 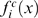 for each phenotype 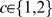. We assume that the gene expression level of gene 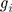 under phenotype 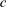 follows a Gaussian distribution with mean 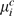 and standard deviation 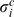. These parameters were estimated based on all available samples 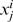 that correspond to the phenotype 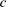. The estimated PDFs can then be used for computing the log-likelihood ratios. In practical applications, we often do not have enough training data for reliable estimation of the PDFs 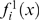 and 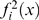. This may make the computation of LLRs sensitive to small changes in the gene expression profile. To avoid this problem, we normalize the 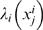 as follows
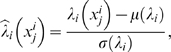
(3)where 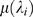 and 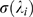 are the mean and standard deviation of 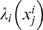 across all samples, respectively. Figure 1 illustrates the overall procedure for inferring the activity of a given pathway.

### Discriminative Power of Pathway Markers

In order to compare the proposed pathway activity inference scheme with other existing methods, we performed the following experiments. In our first experiment, we selected the top 50 differentially expressed pathways using the method proposed by Tian et al. [22]. To assess the ability of a given pathway in discriminating between different phenotypes, Tian et al. computes the 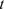-test statistics scores for all member genes and take their average to compute an aggregated score 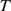 that can serve as an indicator of the pathway's discriminative power. After prescreening the top 50 pathways that have the largest absolute 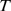 values, we computed the activity score for each of these pathways using the proposed inference method as well as other methods. The obtained pathway activity scores were then used to compute the 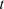-test statistics score for each pathway marker. The 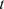-test scores were used to assess the discriminative power of pathway markers and to compare different inference methods.

In this work, we compared five different pathway activity inference methods: the mean and the median methods [25], the PCA-based method [23], [28], the CORG-based method [26], and the inference method proposed in this paper. For the mean, median, and CORG-based methods, we computed the score 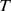 by averaging the 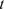-test scores of the expression values of the member genes. For the PCA-based method, we computed 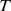 by averaging the *absolute* 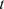-test scores of the gene expression values, since the PCA can naturally combine expression values regardless of whether they are positively correlated or negatively correlated with the phenotype of interest. For our proposed method, we computed 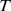 by averaging the 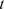-test scores of the LLRs of the member genes, since we estimated the pathway activity score based on LLRs instead of the original expression values.

We also evaluated the robustness of each inference method in identifying good pathway markers, by ranking the pathways using one of the two breast cancer datasets, and then assessing the discriminative power of the pathways based on the other dataset. Again, 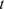-test statistics of the pathway activity scores were used to compare different inference methods.

In our second experiment, we computed the 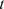-test statistics scores for all 639 pathways without any prescreening, and compared the effectiveness of different pathway activity inference methods based on the computed scores. As in the first experiment, we also evaluated the robustness of each inference method for finding effective pathway markers, by ranking the pathways according to the 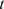-test scores estimated using one of the datasets, and then evaluating their discriminative power on the other dataset.

### Evaluation of Classification Performance

In order to evaluate the classification performance of the proposed pathway activity inference method, we performed the following cross-validation experiments.

**Figure 2:**
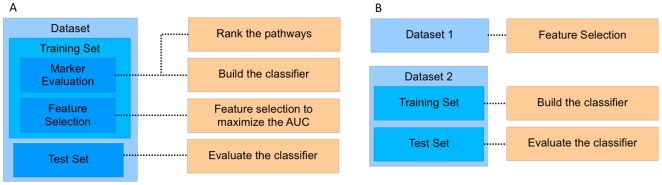
Illustration of the experimental set-up. (A) In the within-dataset experiments, part of the training set, referred as the marker-evaluation set, is used for ranking the pathway markers according to their discriminative power and building the classifier. The optimal set of features are selected based on the remainder of the training set, referred as the feature-selection set. The performance of the resulting classifier is evaluated using the test dataset. (B) In the cross-dataset experiments, one of the datasets is used to find the optimal set of features, and the other dataset is used to build a classifier based on the preselected features and to evaluate the classifier.

For *within-dataset experiments*, the samples in a dataset were randomly divided into five subsets of equal size, where the samples in four of these subsets were used for training the classifier and the remaining subset was used for assessing the classification performance. This has been repeated by using each subset as the test set to obtain more reliable results. The training set was divided again into three equal-sized subsets. Two thirds were used for ranking the pathway markers and building the classifier (the “marker-evaluation” dataset), and one third of the training set was used for feature selection (the “feature-selection” dataset). All samples in the training set were used to estimate the PDFs of the gene expression values under different phenotypes. To build the classifier, we evaluated each pathway based on the discriminative power of its activity score to classify samples. The pathways were sorted in increasing order of the 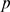-value. After ranking the pathways, we built the classifier, either based on logistic regression or LDA (linear discriminant analysis), as follows. Based on the marker-evaluation dataset, we first constructed the classifier with only one feature, namely, the pathway marker with the lowest 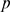-value. The performance of the classifier was then measured by computing the AUC (Area Under ROC Curve) [33] on the feature-selection dataset. Next, we enlarged the set of features by selecting the pathway marker with the lowest 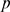-value among the remaining pathways. A new classifier was trained using the selected features on the marker-evaluation dataset and its classification performance was again assessed on the feature-selection dataset. The added pathway marker was kept in the feature set if the AUC increased, and it was removed otherwise. We repeated the above process for all pathway markers to optimize the classifier. The performance of the optimized classifier was evaluated by computing the AUC on the test dataset. These experiments have been repeated for 100 random partitions of the entire dataset. We report the AUC, averaged over 500 experiments, as the overall performance measure of the classification method at hand. The overall process of the within-dataset experiment is illustrated in Fig. 2A.

In order to evaluate the reproducibility of the pathway markers across different dataset, we performed *cross-dataset experiments*, where one dataset was used for selecting the pathway markers, and the other dataset was used for building the classifier based on the selected markers and evaluating its performance. First, we selected the optimal set of features (i.e., pathway markers) based on one dataset, by optimizing the AUC metric. The process for selecting the feature set was similar to the one used in the within-dataset experiments. The samples in the other dataset were divided into five subsets of equal size. Four fifths of samples were used to train the classifier using the selected features, and one fifth of samples were used to evaluate the performance of the constructed classifier. We repeated this experiment by using each of the five subsets as the test set and using the rest for training. The above experiment was repeated for 100 random partitions of the entire dataset, and the average AUC over the 500 experiments was reported as the performance measure. It is important to note that feature selection is performed solely based on the first dataset. During the cross-validation experiments using the second dataset, the training set (that consists of four fifths of samples in the same dataset) is simply used to build the classifier based on the preselected set of features. The overall goal of these cross-dataset experiments is to evaluate the reproducibility of the feature set, selected using the proposed pathway activity inference scheme, across different datasets. Figure 2B illustrates the overall process of the cross-dataset experiment.

To compare the proposed method with other existing methods, we performed the described within-dataset experiments and the cross-dataset experiments using other pathway activity inference methods (mean, median, PCA, and CORG). In addition, we also evaluated the performance of a gene-based classifier that uses individual genes as diagnostic markers, following a similar procedure. In this study, we included the top 50 pathway markers in the initial marker set, which were selected according to the method in Tian et al. [22] as elaborated in the previous subsection. For the gene-based classifier, we included the top 50 gene markers with the lowest 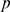-values in the initial marker set, in order to keep the maximum number of features identical.

### Computing the Area under ROC Curve

In this work, we evaluated the performance of a classifier based on the AUC (Area Under ROC Curve). The AUC metric has been widely used for evaluating classification methods, since it can provide a useful summary statistics of the classification performance over the entire range of specificity and sensitivity values. To compute the AUC, we adopted the method proposed in [33]. For a given classifier, let 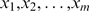 be the output of the classifier for positive samples, and let 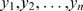 be the output for negative samples. Then, the AUC metric 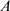 for the classifier is given by:
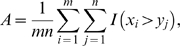
(4)where 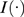 is the indicator function. The AUC is actually the empirical probability that a randomly chosen positive sample is ranked higher than a randomly chosen negative sample. It can be shown that the AUC measure is equivalent to the Mann-Whitney 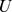-test (also called the Wilcoxon rank-sum test) statistics.

## Results

### Probabilistic Pathway Activity Inference Improves the Discriminative Power of Pathway Markers

**Figure 3:**
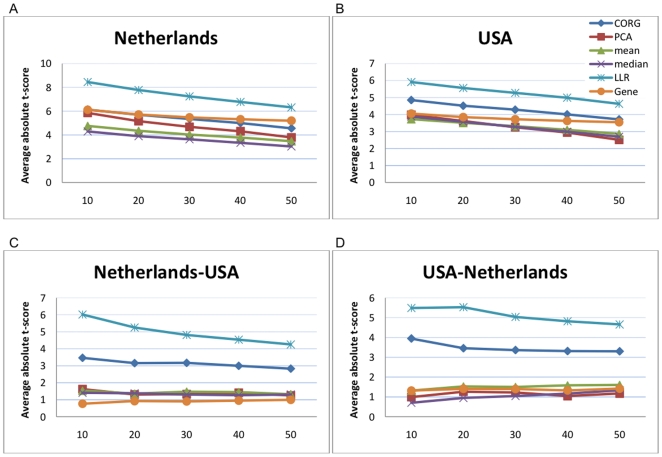
Discriminative power of prescreened pathway markers and single gene markers. (A) Mean absolute 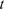-score of the top 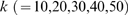 markers for the Netherlands breast cancer dataset. Pathway activities have been inferred using five different methods: CORG, PCA, mean, median, and LLR (proposed method). The discriminative power of the top gene markers was estimated for comparison (labeled as “Gene”). (B) Mean absolute 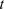-score of the top markers for the USA breast cancer dataset. (C) The markers were ranked based on the Netherlands dataset and the mean absolute 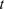-score of the top 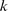 markers was computed based on the USA dataset. (D) The markers were ranked based on the USA dataset and the mean absolute 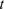-score of the top markers was computed based on the Netherlands dataset.

We evaluated the discriminative power of pathway markers, where the pathway activities were inferred using the proposed method as well as other inference methods. For effective comparison of the proposed inference method with other existing methods, we carried out similar experiments as those performed in [26] to assess the discriminative power of pathway markers. For each breast cancer dataset, we first used the method of Tian et al. [22] to select the top 50 pathways among the 639 pathways obtained from the MSigDB [21] (see Methods). We computed the actual activity scores of the top 50 pathways based on each pathway activity inference scheme, and ranked the pathways according to their discriminative power. Figure 3 shows the discriminative power of the top pathways, where the 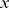-axis corresponds to the number 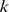 of top pathways that were considered, and the 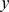-axis shows the mean absolute 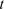-score of the top 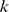 pathways. We compared five pathway activity inference methods, namely, the CORG-based method [26], PCA-based method [23], [28], mean and median methods [25], and the LLR-based method proposed in this paper. For comparison, we also evaluated the discriminative power of the top 50 single gene markers, which were chosen among the 3,271 genes covered by the 639 pathways used in this study. The results obtained from the Netherlands breast cancer dataset [10] and the USA breast cancer dataset [11] are shown in Fig. 3A and Fig. 3B, respectively. As we can see from these results, the proposed pathway activity inference scheme, which computes the pathway activity score by combining the log-likelihood ratios of the member genes, significantly improved the power of pathway markers to discriminate between metastatic samples and non-metastatic samples. Interestingly, the top gene markers often compared favorably to pathway markers. On the Netherlands dataset, the expression levels of the top genes had larger discriminative power than the pathway activity scores inferred by the CORG, PCA, mean, and median methods. Only the pathway activity scores estimated by the proposed method were more discriminative than the gene expression values. On the USA dataset, gene markers were more discriminative than pathway markers based on mean, median, and PCA methods, but less discriminative compared to pathway markers based on the proposed method and the CORG method.

To evaluate the reproducibility of pathway markers, we ranked the markers based on one dataset and evaluated their mean absolute 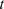-score using the other dataset. Figure 3C shows the result for ranking the markers based on the Netherlands dataset and computing the mean absolute 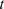-score of the top 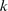 markers using the USA dataset. Similarly, Fig. 3D shows the result for ranking the markers based on the USA dataset and computing the mean score of the top 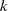 pathways using the Netherlands dataset. These results clearly show that the pathway markers selected based on the proposed inference method retain significantly large discriminative power across different datasets. In fact, in both cross-dataset experiments, the pathway activity scores computed by the LLR method were much more discriminative than the activity scores computed by other inference methods as well as the expression values of the top gene markers. Altogether, these results imply that the proposed method can find better diagnostic markers with higher reproducibility. Also note that the single gene markers, which had considerably large discriminative power within a dataset (see Figs. 3A and 3B), lost most of the discriminative power in a different dataset.

**Figure 4:**
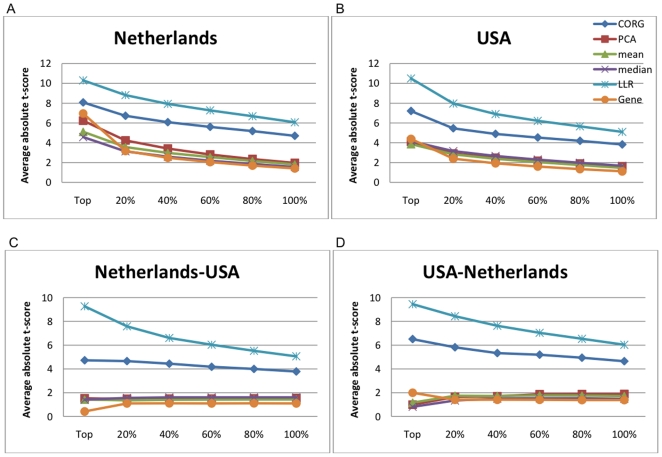
Discriminative power of all pathway markers and gene markers. (A) Mean absolute 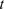-score of the top 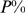 markers for the Netherlands dataset. (B) Mean absolute 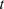-score of the top markers for the USA dataset. (C) The markers were ranked based on the Netherlands dataset and the mean absolute 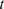-score of the top 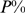 markers was computed based on the USA dataset. (D) The markers were ranked based on the USA dataset and the mean score of the top 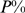 markers was computed based on the Netherlands dataset.

Next, we performed similar experiments for all 639 pathways and all 3,271 genes covered by these pathways, without any prescreening (see Methods). The results of these experiments are shown in Fig. 4, where the 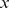-axis indicates the ratio 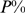 of the top pathways that were used to compute the mean absolute 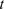-score, and the 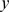-axis corresponds to the estimated mean absolute 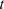-score of the top 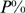 pathways. The discriminative power of the pathway markers and the single gene markers on the Netherlands dataset is shown in Fig. 4A, and the discriminative power of the markers on the USA dataset is shown in Fig. 4B. The results obtained from cross-dataset experiments are summarized in Fig. 4C and 4D. In Fig. 4C, the markers were ranked according to their discriminative power on the Netherlands set, and their mean absolute 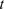-scores were computed using the USA dataset. The results for ranking the markers based on the USA dataset and computing the scores using the Netherlands set are shown in Fig. 4D. All these experiments show that the pathway activity scores measured by the proposed LLR method are much more discriminative than the scores computed by other inference methods and also the expression values of individual genes. Furthermore, we can see that the pathway markers that were chosen based on the LLR-based pathway activity scores are more reproducible and their activity scores retain significant amount of discriminative capability across independent datasets.

### Proposed Pathway Activity Inference Scheme Leads to More Accurate and Reliable Classifiers

We used the proposed pathway activity inference scheme for classification of breast cancer metastasis, to evaluate its usefulness in discriminating different cancer phenotypes. For a fair and effective comparison with other inference schemes, we again adopted a similar experimental set-up that was used in [26] to evaluate the performance of the CORG-based method, a state-of-the-art pathway activity inference scheme that uses only the condition-responsive genes in a given pathway. For each breast cancer dataset, we performed five-fold cross-validation experiments, where four fifths of samples were used for constructing the classifier and the remaining one fifth of samples were used for evaluating the classification performance (see Methods). While constructing the classifier, we used the LLR-based pathway activity inference method for assessing the discriminative power of each pathway marker and selecting the optimal set of markers to be used in the classifier. The constructed classifier also used the pathway activity scores computed by the proposed inference method to distinguish metastatic breast cancer samples from non-metastatic samples. In our experiments, we defined the initial set of pathway markers as the top 50 pathways selected using the method by Tian et al. [22] (see Methods). We assessed the classification performance using the AUC metric. We repeated the five-fold cross-validation for 100 random partitions of the given dataset, and averaged the resulting 500 AUCs to obtain a reliable performance measure of the classification method. To compare the classification performance of different inference methods, we also repeated the previous experiments using the CORG, PCA, mean, and median methods for inferring the pathway activities. For comparison, we also evaluated the performance of the gene-based classification method. We included the top 50 discriminative genes in the initial marker set, to keep the maximum number of features identical for all classification methods.

**Figure 5:**
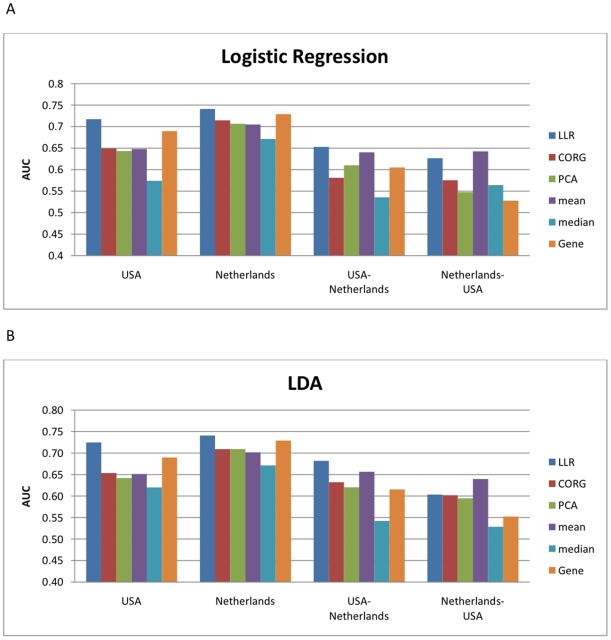
Performance of different classification methods. The bar charts show the average AUCs for different classification methods. Five pathway-based methods that use distinct pathway activity inference schemes (LLR, CORG, PCA, mean, and median) and a gene-based method were compared. (A) Classifiers were constructed based on logistic regression. Results of within-dataset experiments based on the USA and Netherlands datasets are shown in the two charts on the left. The two charts on the right show the results of the cross-dataset experiments. (B) The performance of different classification methods based on LDA (linear discriminant analysis).

Figure 5 summarizes the results of the cross-validation experiments. In the first set of experiments, we used logistic regression for classifying the samples. The classification results of different approaches based on logistic regression are shown in Fig. 5A. The two bar charts on the left of Fig. 5 correspond to the two within-dataset experiments based on the USA breast cancer dataset (labeled as “USA”) and the Netherlands dataset (labeled as “Netherlands”), respectively. In these within-dataset experiments, the initial set of top 50 markers have been selected using the entire dataset, in order to reduce the effect of sensitivity in marker selection when comparing different pathway-based methods. The cross-validation experiments have been performed based on the selected initial set of markers (see Methods). As we can see in these bar charts, the proposed method achieved the highest classification accuracy among all methods, in both experiments. The CORG-based method compared favorably to other pathway-based methods, though outperformed by the proposed method. We can also see that the gene-based classifier performed very well in within dataset experiments, which is not surprising if we consider the high discriminative power of the top gene markers observed in Figs. 3A and 3B.

The results of the cross-dataset experiments are shown in the two bar charts on the right of Fig. 5A. The chart labeled as “USA-Netherlands” shows the results for selecting the features using the USA dataset, and training/evaluating the classifier using the Netherlands dataset. Similarly, the chart labeled as “Netherlands-USA” shows the classification performance for choosing the feature set using the Netherlands dataset, and training and evaluating the classifier based on the USA dataset. As we can see, the proposed LLR-based method outperformed most of the other methods in both cross-dataset experiments. Only the mean-based approach showed better performance than the proposed approach on the Netherlands-USA cross-dataset experiment. These results show that the proposed pathway activity inference method can find a better feature set that is more reproducible across datasets, compared to other activity inference methods. Despite the good performance in within-dataset experiments, gene-based classifiers performed typically worse than many pathway-based classifiers, which shows the poor reproducibility of the feature sets based on individual gene markers.

We also repeated the entire experiments using LDA (linear discriminant analysis), instead of logistic regression, for building the classifiers. The results are shown in Fig. 5B, where we can see similar trends as in Fig. 5A. The proposed classification method yielded the highest classification accuracy in both within-dataset experiments, and it also outperformed other methods in cross-dataset experiments, with the only exception of the mean-based inference method in one of the experiments.

**Figure 6:**
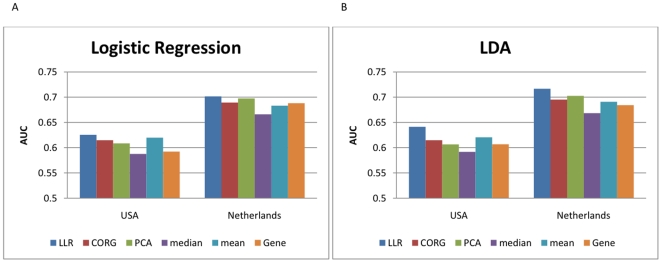
Performance of different classification methods. The bar charts show the average AUCs of within-dataset experiments for five pathway-based methods (LLR, CORG, PCA, mean, and median) and a gene-based method. In these experiments, the top 50 pathways have been reselected in every experiment using the designated training set. (A) Classification results based on logistic regression. (B) Classification results based on LDA (linear discriminant analysis).

Finally, in order to analyze the overall effect of preselecting the initial marker set, we carried out another set of within-dataset experiments, where the initial markers were reselected in every experiment using only the designated training data. The classification results are shown in Fig. 6A and 6B for logistic regression and LDA, respectively. As we can see from these figures, the preliminary marker selection step has important influence on the overall classification results, where the sensitivity of the selection method may adversely affect the performance of the resulting classifiers. However, as we can see from Fig. 6, the relative performance between different classification methods showed similar tendency as in the previous set of experiments (see Fig. 5), and the proposed method consistently outperformed the other methods in all experiments.

### Proposed Method Leads to Robust Classifiers That Yield Symmetric Results for Dataset Inversion

**Figure 7:**
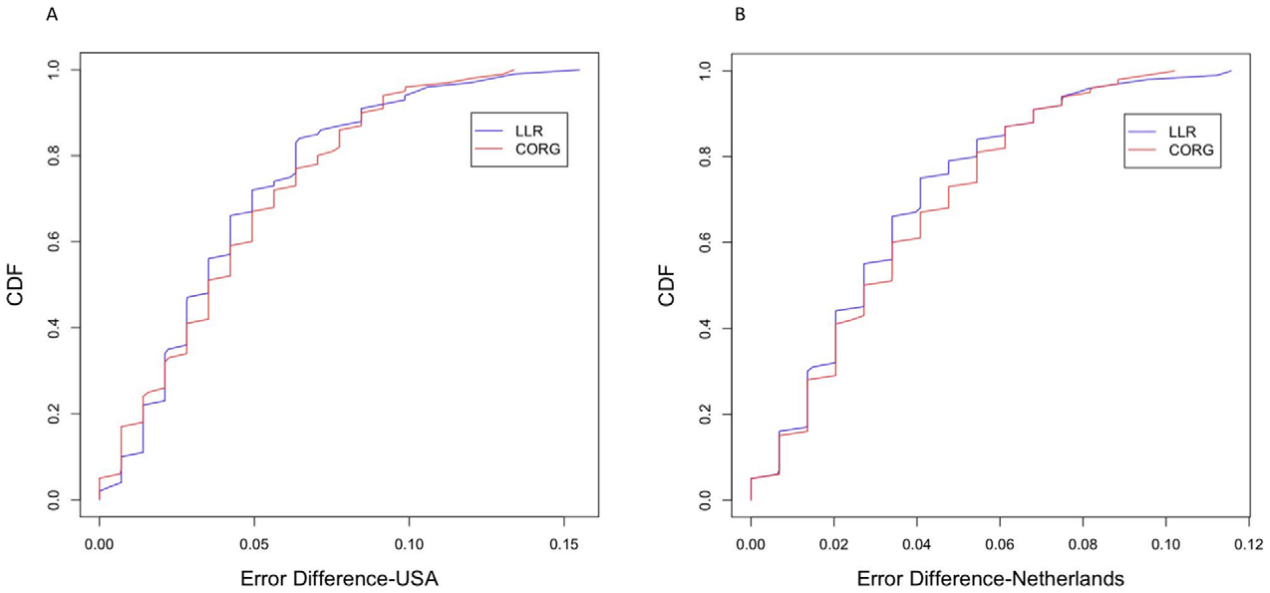
Robustness of the proposed classification scheme. To assess the robustness of the proposed classification scheme, two-fold cross-validation experiments have been performed, where we measured the change in classification error after interchanging the training and test sets. (A) Cumulative distribution of the error difference for the USA dataset. (B) Cumulative distribution of the error difference for the Netherlands dataset.

Ultimately, we want to construct a robust classifier that yields accurate and consistent classification results on independent gene expression datasets. Given two independent datasets of similar size, where one dataset is used for training the classifier and the other dataset is used for evaluation, a robust classification scheme would show consistent classification performance if the training set were interchanged with the test set. However, the USA breast cancer dataset [11] and the Netherlands dataset [10] had been obtained from different microarray platforms and also preprocessed using different methods, which makes it practically difficult to evaluate the robustness of the proposed classification method by training the classifier based on one of the datasets and evaluating its performance on the other dataset. For this reason, we performed the following two-fold cross-validation experiments to assess the robustness of the proposed approach. First, we randomly divided a given dataset into two subsets of equal size. One of the subsets was used to build an actual classifier based on LDA with a classification threshold of 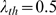. The classifier was then used to classify the samples in the other subset and the classification error rate was computed. Next, we interchanged the training set and the test set and repeated the previous experiment. In order to find out whether we can obtain consistent classification performance after interchanging the training and test sets, we computed the absolute difference between the two classification error rates. We repeated this experiment for 250 random partitions of each breast cancer dataset, and estimated the distribution of the absolute error difference. For comparison, we carried out the above experiments using the proposed pathway activity inference scheme as well as the CORG-based scheme [26]. The proposed classification scheme resulted in a relatively small average error difference of 0.0414 on the USA dataset, and 0.0324 on the Netherlands dataset. The CORG-based classification scheme yielded a slightly higher error difference, whose average was 0.0429 for the USA dataset and 0.0345 for the Netherlands dataset. Figure 7 shows the cumulative distribution of the classification error difference on the two datasets for the respective methods. These results indicate that both pathway-based classification schemes can lead to the construction of robust classifiers that yield consistent results on different datasets, where the proposed scheme compares favorably to the CORG-based scheme.

## Discussion

In this paper, we have proposed a novel probabilistic pathway activity inference scheme that estimates the activation level of a pathway based on the log-likelihood ratios (LLRs) of the member genes. The proposed method can effectively address several shortcomings of the previous pathway activity inference methods, thereby improving the discriminative power of the pathway markers. For example, the methods proposed by Guo et al. [25] estimate the pathway activity by taking the mean or median of the gene expression values of the member genes. These methods cannot effectively capture the coherent gene expression patterns that may be present within a pathway. For example, suppose a member gene is positively correlated with a phenotype of interest, while another gene in the same pathway is negatively correlated with the given phenotype. In this case, we may lose much of the discriminative information contained in the respective gene expression values if we average them out. The PCA-based inference method used in a number of studies [23], [28] can somewhat relieve this problem. In the PCA approach, the first basis vector captures the average expression pattern of the member genes, and the first principal component can estimate the presence and the strength of this pattern in a gene expression profile. However, not all the member genes may alter their expression levels under different phenotypes in a consistent manner. In fact, some genes may have expression changes that are irrelevant to the phenotypic change of our interest. To address this problem, Lee et al. [26] proposed a new pathway activity inference method that uses only a subset of member genes, called CORGs (condition-responsive genes), whose combined expression levels are highly discriminative of the phenotypes. However, the CORG-method may disregard member genes that have consistent, but not large, expression changes under different phenotypes.

The proposed LLR-based method provides an effective solution to these problems. First of all, by using the LLR of a member gene, instead of directly using its expression value, the proposed method can capture the consistent gene expression changes that are related to the phenotypic change. Moreover, since the LLR is computed based on the difference in distribution of the gene expression values under different conditions, the direction and the amount of expression changes do not have large effects on the overall discriminative power of the pathway marker. Furthermore, the proposed method fully utilizes the available discriminative information in all the member genes, not just some of them; and it naturally weights and combines the support from each member gene in a given pathway to increase the discriminative power of the corresponding pathway marker. As we have demonstrated in this paper, the LLR-based pathway activity inference scheme significantly improves the discriminative power of the pathway markers, increases the overall classification accuracy, and finds reliable pathway markers that are more reproducible across different datasets. Therefore, the proposed method may ultimately lead to the construction of more reproducible classifiers. The two-fold cross-validation experiments, where we measured the change in classification error that resulted from interchanging the training and test sets, demonstrated the potential of the proposed scheme for building robust and reproducible classifiers.

Currently, one limitation of the pathway-based classifiers is the limited coverage of genes by known biological pathways. We believe that the classification performance of the pathway-based methods will be considerably improved once we have a more complete list of biological pathways. One possible way to overcome this problem is to identify effective pathway (or subnetwork) markers by overlaying a protein-protein interaction (PPI) network with gene expression data and searching for significantly differentially expressed regions in the given network, as proposed in [34]. In this work, we assumed that the expression values of a gene follows a Gaussian distribution. Although this has been shown to be a good approximation in our experiments, using alternative distributions that better fit the expression data may further improve the overall classification performance. For example, we may consider using gamma distributions as proposed by Efroni et al. [35].
